# Neonatal Sepsis-Induced Coagulopathy in the Light of Developmental Hemostasis: Friend or Foe?

**DOI:** 10.3390/medicina62030584

**Published:** 2026-03-20

**Authors:** Paraskevi Papadogeorgou, Rozeta Sokou, Sotirios P. Fortis, Vasiliki Mougiou, Theodora Boutsikou, Nicoletta Iacovidou, Serena Valsami

**Affiliations:** 1 Neonatal Department, Aretaieio Hospital, Medical School, National and Kapodistrian University of Athens, 11528 Athens, Greece; paraskevipapadogeorgou@gmail.com (P.P.); sokourozeta@gmail.com (R.S.); vassouli@hotmail.com (V.M.); theobtsk@gmail.com (T.B.); niciac58@gmail.com (N.I.); 2 Department of Neonatology, General Hospital of Athens “Alexandra”, 11528 Athens, Greece; 3 Laboratory of Reliability and Quality Control in Laboratory Hematology (HemQcR), Department of Bio-Medical Sciences, School of Health & Caring Sciences, University of West Attica (UniWA), 12243 Athens, Greece; sfortis@uniwa.gr; 4 Laboratory of Hematology and Blood Bank Unit, Aretaieio Hospital, Medical School, National and Kapodistrian University of Athens, 11528 Athens, Greece

**Keywords:** coagulation, neonates, developmental hemostasis, sepsis, sepsis-induced coagulopathy

## Abstract

The concept of ‘developmental hemostasis’ from birth to infancy and onwards to childhood and adulthood was introduced in the 1980s and is used to indicate the fundamental discrepancies of hemostatic mechanism between children and adults. The underlying differentiations are more pronounced in term and even more in preterm neonates. Hemostatic alterations tend to improve throughout childhood and adolescence but still imply a great example of the basic concept that children do not simply represent small adults. Many neonatal coagulation disorders lead to severe morbidities, such as intraventricular hemorrhage and intracerebral infarct, with critical consequences on long-term neurodevelopmental outcome. As the limits of viability have decreased and many preterm and severely affected neonates survive and grow up, a broad understanding of hemorrhagic and thrombotic complications in neonates is very important, in order to provide prompt identification and treatment. Coagulation abnormalities are usually induced by specific pathophysiologic disorders, and neonatal sepsis is a significant trigger of hemostatic derangement. Despite the initial protective role of coagulation activation during the early stages of sepsis, ultimately hemostatic abnormalities exert a substantial impact on clinical outcome and prognosis. This review explores developmental aspects of coagulation, particularly in relation to neonatal sepsis.

## 1. Introduction

Coagulation is the dynamic balance between the procoagulant pathway that leads to clot formation and the anticlotting mechanism that inhibits clot propagation beyond the injury site. However, this balance may be disrupted, either towards bleeding or thrombosis [1].

The objective of this review is to clarify the main aspects of clotting and anticlotting mechanisms in neonates, as well as developmental hemostasis and its evolutional significance during early human development. Moreover, an attempt has been made towards a thorough literature update on sepsis-induced coagulopathy (SIC), a topic with major clinical significance and impact in the field of neonatology. Although the review is narrative, rather than systematic, great attention has been paid in the detailed search of the most recent and relevant publications, in order to synthesize a comprehensive and highly informative review on the topic. The fact that recent studies on neonatal hemostasis are lacking highlights the scientific and clinical importance of our review and the necessity of relevant studies to be conducted and published. The search process was mainly based on Pubmed-indexed publications, while this review represents a complementary project in previously presented research on coagulation abnormalities in neonates with sepsis and/or congenital heart disease [2,3]. The language of publications was restricted to English. Original research studies, review articles, letters to the editor, randomized controlled trials (RCTs), non-RCTs, case reports, case series, and cohort studies were included. The review has been outlined in three major axes: First, a general overview of the coagulation process, anticlotting mechanism and fibrinolysis is presented. Next, the main concepts of developmental hemostasis are integrated. Finally, a thorough analysis of neonatal SIC is performed.

The coagulation system involves primary and secondary hemostasis, as well as fibrinolysis [4].

### 1.1. Primary Hemostasis

Primary hemostasis represents the initial “platelet plug” formation, as a result of complex interactions between platelets, the vessel wall and adhesive proteins. Normally platelets do not adhere to intact vascular endothelium. However, endothelial injury leads to secretion of von Willebrand factor (VWF), a multimeric protein that serves as a bridge between endothelial collagen and platelet surface receptors leading to platelet adhesion and aggregation. Following adhesion, platelets form numerous pseudopods, their surface area increases [5], and various factors are released, such as calcium and thromboxane A2 (TxA2), which stimulate aggregation of platelets and clotting factors [6]. The endpoint is the platelet plug formation, which seals off vascular injury temporarily and is subsequently stabilized by deposition of fibrinogen and later converted to fibrin. von Willebrand factor (VWF) is released at sites of vascular injury as an unusually large multimer (ultra-large VWF, ULVWF), the hyperactive and highly thrombogenic form of VWF. The size of ULVWFis downregulated by ADAMTS-13 (a disintegrin and metalloprotease with thrombospondin type-1 motives), the cleaving protein of ULVWF, which is released by the endothelium [7,8].

### 1.2. Secondary Hemostasis

Traditionally, secondary hemostasis is classified into an intrinsic and an extrinsic pathway. Although this principle provides a useful explanation of the in vitro coagulation tests, it lacks the ability to explain certain clinical scenarios, such as hemophilia type A and B. Classical aspects of coagulation fail to clarify why the defective intrinsic pathway in hemophiliac patients is not bypassed by the integral extrinsic pathway.

A revised cell-based coagulation model was adopted, stating that the intrinsic pathway is not a parallel pathway, but instead it amplifies thrombin, primarily produced by the extrinsic pathway [9]. Coagulation is initiated by tissue factor (TF) expression at the site of vascular injury. Tissue factor (TF) binds to activated factor VII (FVIIa) to activate factor IX (FIX) and factor X (FX). Activated factor X (FXa) activates factor V (FV) to activated factor V (FVa), and together they form the prothrombinase complex, which converts prothrombin or factor II (FII) to thrombin (activated FII, FIIa). The initiation phase is probably constantly active, leading to constant production of thrombin in small quantities, irrespectively of vascular injury. The next phase is the amplification phase, activated only after vascular injury and characterized by thrombin’s action as positive feedback for activation of platelets, as well as FV, FIX, factor XI (FXI) and unbinding of factor VIII (FVIII) from VWF. Furthermore, factor XII (FXII) is exposed, at sites of vessel wall injury, to collagen and subendothelial connective tissue and is activated to activated FXII (FXIIa), further catalyzing the conversion of FXI to activated FXI (FXIa). Activated factor XI (FXIa) activates FIX to activated FIX (FIXa), which in turn activates FX to FXa. Concurrently, FXIIa initiates the process of fibrinolysis. The next phase is the propagation phase, during which the accumulated enzyme complexes on the platelet surface support further generation of thrombin and subsequently fibrin, leading to a firm and large clot. Thrombin activates factor XIII (FXIII) (fibrin stabilizing factor), which links fibrin polymers and ensures a stable platelet plug. In addition, thrombin activates thrombin-activatable fibrinolysis inhibitor (TAFI), an essential factor in protecting the clot from fibrinolysis [10,11]. In addition, thrombin binds to thrombomodulin (TM), an endothelial cell receptor, leading to the activation of the natural coagulation inhibitor protein C (PC) [12].

### 1.3. Anticlotting Mechanisms

Excessive activation of the coagulation cascade is halted by natural inhibitors, such as antithrombin (AT), protein C and protein S (PC and PS, respectively). The first step of the termination process is the release of tissue factor pathway inhibitor (TFPI) from endothelial cells and platelets. Antithrombin is a non-vitamin-K-dependent glycoprotein that inhibits mainly thrombin and FXa and less importantly FIXa, FXIa and FVIIa. Heparin acts as an anticoagulant by enhancing the activity of AT by at least 1000 times. Besides AT, thrombin is inhibited by heparin cofactor II (HCII), α2-macroglobulin and a1-antitrypsin [12].

Protein C (PC) is a vitamin-K-dependent natural inhibitor which is converted to activated protein C (APC) by thrombin. Activated protein C (APC) inhibits coagulation by degrading FVa and activated FVIII (FVIIIa) and also has anti-inflammatory and anti-apoptotic effects. Protein S (PS) serves as its cofactor. Finally, the most recently identified component of the anticoagulant system is protein Z (PZ)-dependent protease inhibitor (ZPI), which inhibits FXa [13].

### 1.4. Tertiary Hemostasis or Fibrinolysis

Fibrinolysis is activated in parallel to activation of coagulation cascade and exerts a pivotal role in limiting the clot’s size. The process of fibrinolysis is initiated by the attraction of plasminogen and tissue plasminogen activator (tPA) to the lysine residues of fibrin. Plasminogen is then converted to plasmin by tPA. Plasmin dissolves the fibrin clot into soluble fragments, including D-dimers. This reaction is catalyzed by tPA and urokinase plasminogen activator (uPA), produced by the vascular endothelium [4].

**Figure 1:**
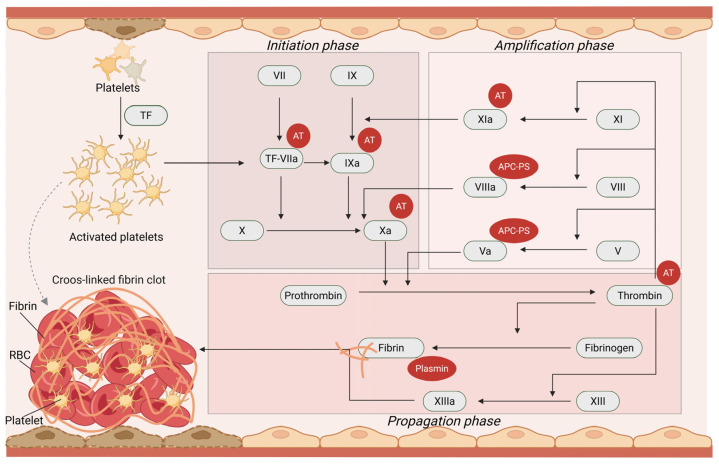
A graphical outline of the cell-based coagulation model. Endothelial damage leads to platelet adhesion, activation and aggregation, while TF expressed at the site of vascular injury binds to FVIIa to activate FIX and FX. Activated FX (FXa) activates FV to FVa, and together they form the prothrombinase complex, which converts prothrombin to thrombin (initiation phase). Thrombin acts as positive feedback for activation of platelets, FV, FIX, FXI and unbinding of FVIII from VWF (amplification phase). The accumulated enzyme complexes on the platelet surface support further generation of thrombin and subsequently fibrin, leading to a firm and large clot (propagation phase). Thrombin activates FXIII (fibrin stabilizing factor) which links fibrin polymers and ensures a stable platelet plug. Anticlotting and fibrinolytic mechanisms are activated in parallel, as demonstrated by factors in red circles. Abbreviations: APC, activated protein C; AT, antithrombin; PS, protein S; RBC, red blood cell; TF, tissue factor. Created in BioRender. Fortis, S. (2026) https://BioRender.com/0xvyj5u. Agreement number: ZH29GQW2HG.

Widespread fibrinolysis is prevented by plasmin’s inhibitors, such as α2-antiplasmin and α2-macroglobulin. Furthermore, the main physiological inhibitor of fibrinolysis is plasminogen activator inhibitor (PAI), which acts by irreversible inhibition of tPA and uPA. Finally, it decreases the affinity of plasminogen to fibrin and reinforces the action of α2-antiplasmin [14]. The principal aspects of clotting and anticlotting mechanisms, as well as fibrinolysis, are depicted in Figure 1.

## 2. Developmental Aspects of Coagulation

The hemostatic mechanism is an evolving process throughout life, and the understanding of physiological age-dependent changes of clotting and anticlotting factors is crucial for the accurate diagnosis of coagulation abnormalities, especially in infancy and childhood.

### 2.1. Peculiarities of Procoagulant Factors in Neonates

Procoagulant factors do not cross the placenta, and the fetus produces them at measurable levels by 10 weeks of gestation. Functional clot formation and lysis can be detected even earlier, by 8 weeks of gestation. Certain coagulation parameters exhibit unique fetal characteristics, and the fetal and neonatal clot is more translucent relative to healthy adults, with reduced fibril length and prolonged clotting time at lower pH [12]. Although further functional and quantitative maturation occurs throughout gestation, and especially after birth, neonates demonstrate lower levels of many coagulation parameters. Vitamin-K-dependent factors (FII, FVII, FIX, and FX) are approximately at 50% of adult values in newborns. This was initially described by Andrew et al. in the late 1980s but was also verified by newer studies using modern technology and reagents, as reported by Monagle et al. [15,16]. What is more, contact system factors (FXI, FXII, prekallikrein (PK) and high-molecular-weight kininogen (HMWK)) are also found at lower levels at the time of birth and during the neonatal period. On the other hand, plasma levels of FVIII and VWF in neonates are close to or even higher than adult levels; thus, if a neonate demonstrates low levels of FVIII or VWF, the baby should be evaluated for a deficiency, rather than waiting for normalization at an older age [17]. Besides higher levels of VWF in neonates, a special fetal form of VWF is demonstrated, characterized by ULVWF, probably due to lower levels of ADAMTS-13 [18]. Higher levels of VWF antigen and activity found in early infancy reach a nadir at around 12 months of age and finally increase to adult levels. This last trend is less distinct in individuals with blood group O, who have steadily lower levels of VWF antigen and activity, as well as FVIII [19]. Factor V (FV) and FXIII levels in newborns are comparable to those observed in older children and adults. Fibrinogen is the final substrate in clot formation, leading to the production of an insoluble clot, and it also functions as an acute-phase reactant. Its levels may increase in response to various inflammatory stimuli and may also be influenced by seasonal variation, with higher levels observed during winter. Although fibrinogen concentrations in neonates may be relatively elevated, it is typically present in a “fetal,” functionally impaired form characterized by increased sialic acid content [17].

### 2.2. Neonatal Platelets

As mentioned above, platelet activation is the principal step in the coagulation process. The platelets of preterm and term neonates are structurally like those of adults, but they demonstrate significant functional differences. Neonatal platelets are hyporeactive in response to multiple agonists in vitro, and this hyporeactivity is more pronounced in preterm infants [17]. Neonatal platelets also have an altered transcriptome, resulting in modified protein synthesis and processing, and impaired calcium transport/metabolism and cell signaling. This variation in the platelet transcriptome in association with a deficiency of platelet receptors may be the underlying pathophysiologic mechanism of platelet hyporeactivity in neonates [20,21]. Platelets of preterm neonates are even more hyporeactive than those of full-term neonates, due to lower expression of glycoproteins (GP) and P-selectin on their surface [22,23]. Platelet functional problems are overcome over time, but for how long this hyporeactivity lasts remains unclear [20,24]. It is assumed that adult levels of platelet reactivity are reached on the 5th–9th day of life [25]. Platelet function tests, such as bleeding time and the platelet function analyzer, are not impaired in neonates, while viscoelastic assays demonstrate stronger clot firmness in neonates compared to adults [26,27,28]. Higher levels of VWF, high hematocrit and mean corpuscular volume of red blood cells (MCV) and lower levels of natural anticoagulants in neonatal blood seem to compensate both the neonatal platelet hyporeactivity as well as the decreased coagulation factor levels, resulting in a dynamic equilibrium of the neonatal hemostatic system [15,26,29]. Moreover, platelets are not solely involved in coagulation but also play important roles in angiogenesis, inflammation, and immune system function [30,31].

### 2.3. The Neonatal Anticlotting Mechanism

Newborn infants demonstrate lower levels of anticlotting factors, particularly AT, PC, PS and HCII, in comparison to adults. Antithrombin (AT) is lower during the newborn period and reaches adult levels later in childhood. Concentrations of PC and PS are also lower in neonates and remain reduced throughout childhood in comparison to adults [15,32]. Furthermore, certain animal studies have shown a special fetal form of PC [33]. Although PS levels are markedly lower in newborns, there is no functional impact, due to lacking C4, the binding protein of PS, resulting in the predominance of the free and active PS form. The lower levels of AT, PC, and PS at birth may be compensated for by α2-macroglobulin, another potent anticoagulant present at increased concentrations in neonatal plasma [34]. Furthermore, an additional anticoagulant factor is present in cord blood that inhibits thrombin, similar to HCII. A fetal proteoglycan produced by the placenta is also detectable in the maternal circulation; however, the duration of its persistence in neonatal plasma remains unknown [35,36]. Finally, total TFPI levels in neonates are comparable to those of older children and adults, although the free form of TFPI is substantially reduced [37].

### 2.4. The Fibrinolytic System in Neonates

At birth, plasminogen levels are approximately 75% lower in preterm neonates and 50% lower in full-term neonates compared to adults, reaching adult values by 6 months of age [19,38,39,40]. The levels of α2-antiplasmin at birth are 80% of adult levels, which are attained within the first week of life, indicating an activation of the fibrinolytic system soon after birth [38]. Neonatal plasminogen is functionally immature in neonates, demonstrating slow activation by its major activator, tPA. It has been reported that five times more tPA is required for activation of plasminogen to plasmin in neonates in comparison to adults [41]. In contrast, PAI levels at birth are similar to or even higher than those observed in adults. Reduced levels of plasminogen and tPA, together with elevated PAI concentrations, could theoretically result in suppressed fibrinolytic activity in neonates. However, this effect is mitigated by several compensatory mechanisms, including reduced inactivation of plasmin by α2-antiplasmin. Moreover, fibrinolysis is primarily regulated by endothelial cells through the release of tPA and PAI; therefore, baseline concentrations of fibrinolytic factors may not accurately reflect activity following fibrinolytic activation.

Additionally, α2-macroglobulin appears to play an important role in neonatal fibrinolysis, as its levels during the neonatal period are nearly twice those observed in adults. This increase contributes to the regulation of fibrinolytic activity and helps compensate for decreased plasminogen and tPA levels, as well as elevated PAI concentrations, by limiting plasmin inactivation through alternative inhibitory pathways [42]. Taken together, these findings indicate that fibrinolytic activity in neonates is preserved and functionally adequate [38].

D-dimer measurement is a specific indicator of fibrin lysis by plasmin and reflects coagulation activation, including disseminated intravascular coagulation (DIC) [43]. D-dimer levels are elevated in cord blood samples in healthy neonates, due to activation of the coagulation system during delivery, and are 6–8-fold higher in comparison to adults and remain increased throughout the first year of life [19,44]. Elevated D-dimer levels are observed in conditions such as thromboembolism, tissue injury, infection, malignancy, and hypoxia. However, specific cut-off values for the exclusion of thromboembolism have not been established in neonates, which poses significant diagnostic challenges. Adult reference ranges are not applicable to the diagnosis of thromboembolism or DIC in neonates and children. Diagnosis should rely on clinical findings, imaging for thrombosis, and assessment of platelet counts and coagulation factors for DIC [45].

### 2.5. Prematurity-Related Differences in Hemostatic Mechanism

The aforementioned alterations of clotting and anticlotting factors are more pronounced in neonates born preterm, and the magnitude of change is proportional to the grade of prematurity [16,46,47,48,49]. The levels of vitamin-K-dependent factors are measured to be 30% of adult levels at 24–29 weeks of gestation, while contact factors are similarly reduced [50]. Clotting factors reach their lowest levels at 24–27 weeks of gestation and increase progressively thereafter [48]. Similarly, clotting times are prolonged in preterm neonates compared to full-term neonates, and the prolongation correlates with the degree of prematurity [51,52,53]. As mentioned above, platelets in preterm neonates demonstrate greater hyporeactivity in comparison to term neonates. Particularly, Platelet Glycoprotein (GP) GPIIb/IIIa receptor (integrin αIIbβ3) activation is decreased, binding to fibrinogen is also reduced, and so is platelet degranulation [54,55]. Although the coagulation system in preterm neonates is immature compared with that of full-term infants, their hemostatic function remains effective and adequate [56].

### 2.6. The Impact of Developmental Hemostasis on Laboratory Tests

Lower concentrations of coagulation factors result in prolongation of baseline coagulation assays, particularly Activated Partial Thromboplastin Time (APTT) and Prothrombin Time (PT). Neonates exhibit mildly prolonged PT and APTT compared with adult reference ranges. These values gradually shorten, reaching adult levels by the first week of life (PT) and by 6 months of age (APTT). Age-adjusted normal ranges are fundamental to interpret laboratory results in such patients. Furthermore, hemostatic protein levels cannot clearly predict the clinical phenotype [50,57]. Viscoelastic assays such as thromboelastography (TEG) and rotational thromboelastometry (ROTEM) are influenced by developmental hemostasis. In healthy neonates, coagulation initiation is accelerated compared to children and adults, likely due to elevated concentrations of procoagulant factors such as fibrinogen, FV, FVIII, VWF and FXIII, as well as reduced levels of natural anticoagulants [58,59,60,61,62]. In regard to clot strength, several studies have yielded conflicting results. Several studies have demonstrated reduced clot strength in healthy neonates compared with adults, likely due to impaired fibrin polymerization secondary to fibrinogen immaturity. Platelet functional abnormalities, as well as lower levels of vitamin-K-dependent and contact factors, represent additional contributing factors [60,63]. However, other investigators have reported increased clot strength in neonates compared with older children [27,59]. The discrepancy noted may derive from utilization of different assays. Fibrinolysis as assessed by TEG and ROTEM is enhanced in healthy neonates, probably as a result of altered regulating proteins in this particular age group [59,61,62]. Furthermore, Sokou et al. demonstrated increased fibrinolytic activity in preterm neonates in comparison to full-term neonates, while Motta et al. demonstrated enhanced fibrinolysis in early-preterm neonates in comparison to moderate- and late-preterm neonates [64,65]. Respiratory Distress Syndrome (RDS) is a common morbidity of prematurity and is associated with activation of both coagulation and fibrinolysis in adults and probably preterm neonates [65,66]. However, a hypocoagulable tendency was found in preterm and full-term neonates with RDS compared to healthy neonates in the study conducted by Katsaras et al. [67]. Additionally, studies have yielded conflicting results regarding viscoelastic assays in term and preterm neonates. Strauss et al., as well as Rafaelli et al., demonstrated reduced clot strength in preterm neonates compared with full-term neonates and adults, while this hypocoagulable state resolves over the first month of life [60,68].

Despite the increasing evidence favoring the usefulness of viscoelastic assays in clinical practice of neonatology, several discrepancies exist between the available studies, rendering their application challenging. The underlying etiology of this disparity is multifactorial. First of all, the hemostatic mechanism, as well as platelets, demonstrates gradual quantitative and qualitative maturation postnatally [24,69]; thus, the time of sampling poses a significant influence on the extrapolated results. Besides the maturation of the coagulation system, the clinical condition of patients differs between distinct time points. The first postnatal days are the most critical, associated with great respiratory and hemodynamic instability and particular clinical conditions, such as RDS and Patent Ductus Arteriosus (PDA). The impact of these morbidities and their targeted treatment, such as mechanical ventilation, inotrope administration and ibuprofen on viscoelastic assays, though not thoroughly studied, cannot be overlooked. Moreover, subclinical pulmonary inflammation is more evident during the recovery phase of RDS, favoring hypercoagulability [56,70,71]. Hematocrit (Ht) also interacts with viscoelastic tests, and high Ht is associated with a hypocoagulable state [56,60,72,73]. The majority of studies do not take into account the impact of Ht, while higher Ht values during the first days of life also enhance the significance of time sampling. What is more, gestational age represents a consequential factor. Many studies have included preterm neonates; however, the span of prematurity nowadays extends from 22 weeks to 36^+6^ weeks, with significant differentiation in the hemostatic mechanism per se, as well as the associated morbidities. Thus, it seems reasonable that studies with dissimilar samples in regard to gestational age yield conflicting results. Furthermore, maternal factors may also impair the viscoelastic tests and sometimes may be underestimated, while the origin of blood samples is very important, as a discrepancy has been demonstrated between umbilical cord and venous blood samples of the same neonates [74]. Particular disparities in the study design, patient inclusion criteria, the viscoelastic tests performed and the reagents used are additional factors contributing to the discordance between study results [75]. Nevertheless, the dynamic status and complexity of the hemostatic mechanism is well recognized during the entire life span and particularly during the vulnerable newborn period, implying a significant impact on the laboratory assessment of it. The routine employment of viscoelastic tests in everyday clinical practice is costly, while standardized equipment and special training on assay performance and results interpretation are mandatory [76].

The study by Theodoraki et al. demonstrated that clinically stable preterm neonates between days 5 and 10 of life exhibit shorter clotting time (CT) and clot formation time (CFT) and increased clot formation speed and strength compared with term neonates. Changes in the hemostatic profile of preterm neonates during the first days of life have been well documented and are mainly attributed to the ongoing maturation of the hemostatic system. Although platelet count and function, as well as levels of hemostatic proteins, are reduced early after birth, gradual improvement is observed by the 10th day of life. In parallel, additional factors such as the inflammatory response associated with prematurity may further influence hemostasis, reinforcing a prothrombotic tendency through effects on platelet activity and fibrinogen synthesis and function. Fibrinolytic activity was also increased in preterm neonates compared to full-term neonates. Furthermore, the hypercoagulable state was positively correlated with RDS background, probably due to activation of coagulation and fibrinolysis and fibrin deposition in RDS cases, as mentioned above [56].

Additional evidence confirms the significance of viscoelastic tests in identification of acquired coagulation problems in neonates, as well as in guiding the prompt transfusion of blood products [77]. Studies have shown that ROTEM is a valuable tool for early risk stratification of morbidity and mortality in critically ill neonates, particularly through EXTEM clot formation and clot amplitude parameters [78,79,80,81,82]. In addition, ROTEM can be used to predict bleeding risk in neonates and has the potential to further contribute to individualized transfusion guidance [83,84,85,86]. Thromboelastography can also serve as a valuable tool for guiding fresh-frozen plasma transfusion in neonates undergoing surgery [87]. Nevertheless, data extrapolated from studies should be interpreted with caution due to small sample sizes in certain studies and heterogeneity in laboratory assays and agents used.

### 2.7. Evolution of Hemostatic Mechanism in Childhood

The hemostatic mechanism gradually matures quantitatively and qualitatively. First, the stress of birth upregulates the neonatal coagulation system at the time of parturition. Several coagulation factors are at lower levels in fetuses, as compared to values measured postnatally in preterm and full-term neonates. Furthermore, the mode of delivery should always be taken into account, since certain procoagulant factors, such as FVIII, VWF, FIX, FXI, FXII and plasminogen are found at higher levels in cord blood samples following vaginal delivery vs. elective caesarian section [88]. Most coagulation factors reach adult levels at approximately 6 months of age. In regard to preterm neonates, accelerated maturation takes place, so coagulation parameters also reach adult levels by 6 months of age [15,46].

Although developmental hemostasis is well established, the underlying rationale has not been fully elucidated. First, developmental hemostasis may be absolutely necessary for organogenesis. Specific coagulation proteins, such as TF, TFPI, FV, prothrombin and AT, are essential in embryonic and fetal development, and deletion of the relevant genes is lethal, as demonstrated by animal experiments. Furthermore, TF plays a key role in tissue proliferation and differentiation, supported further by higher concentrations and increased distribution of TF in several tissues in early development in comparison to adults [12]. Studies based on cell cultures have demonstrated that contact factors exert extra properties, such as upregulation of cell proliferation and support of postnatal angiogenesis by FXII and PK [89].

Another explanation may be that the hemostatic system is well balanced and protected against both bleeding and thrombosis early in life, whereas aging exerts a cumulative negative effect [90]. However, developmental aspects of coagulation may be unrelated to the hemostatic process itself and instead reflect the multiple biological functions of clotting and anticoagulant factors, including roles in angiogenesis, inflammation, and wound repair. These alterations may, in turn, trigger additional compensatory mechanisms to maintain hemostatic balance. A well-studied example is AT, which exhibits anti-inflammatory, antiviral, and strong anti-angiogenic properties in addition to its anticoagulant activity [13,91]. What is more, heparin, a potent amplifier of AT’s action, also exerts an anti-angiogenic effect and has been implemented in anti-cancer therapy [92]. Additionally, AT replacement therapy early in life has been shown to be deleterious, associated with increased mortality, possibly due to the anti-angiogenic role of AT, although the exact underlying mechanism has not been fully elucidated [93]. Lower AT levels during fetal life and early infancy may serve as a controlling factor of angiogenesis and are vital for human development. Reduced concentrations of AT during the first months of life are probably compensated by increased α2-macroglobulin levels [16,92]. Furthermore, age-related changes in protein concentrations are not limited to the coagulation system but represent part of a broader physiological phenomenon. Indeed, several plasma proteins were measured in blood samples from healthy neonates on days 1 and 5 of life and from adults, revealing significant differences in the number and abundance of numerous proteins across age groups [94].

Lisman et al. demonstrated that children who received liver transplants from adults continued to have coagulation proteins at levels consistent with their age post-surgery, rather than at adult levels [95]. The regulation of coagulation proteins is likely extrinsic to the liver and may involve the vascular endothelium, whose function is closely linked to coagulation, as demonstrated in DIC. Moreover, altered gene regulation and post-translational modifications affecting protein function, circulation, and clearance may represent additional mechanisms underlying developmental hemostasis [92]. Increased basal metabolic rate in young individuals may also lead to accelerated elimination of proteins from the body [96]. The different age-related values of various procoagulant and anti-coagulant factors result in a hemostatic “equilibrium”, and the changing coagulation system throughout life should be considered physiologic, protected from thromboembolic disease without increased bleeding tendency [92]. Indeed, patients with inherited thrombotic disorders, such as congenital AT deficiency, typically do not develop thrombosis until early adulthood. Elevated α2-macroglobulin evels in children likely constitute an underlying protective mechanism in patients with AT deficiency, who tend to manifest thrombotic complications during adolescence or later, when α2-macroglobulin levels normalize to adult values [97]. Acquired risk factors also pose much smaller thrombotic risk in children than adults. For example, nephrotic syndrome in adults is associated with thrombosis in 20% of cases, in contrast to only 2% in children [92]. Additionally, children rarely have thromboembolic complications after surgery or prolonged immobilization. The molecular basis of this apparent protection has yet to be elucidated [98,99].

Despite developmental hemostasis, the equilibrium is maintained in healthy term neonates because the lower values of procoagulant factors are counterbalanced by the decreased levels of anticlotting proteins. Thus, healthy term neonates do not have increased bleeding or thrombotic risk [25,100].

## 3. Coagulation in Neonatal Sepsis

**Figure 2:**
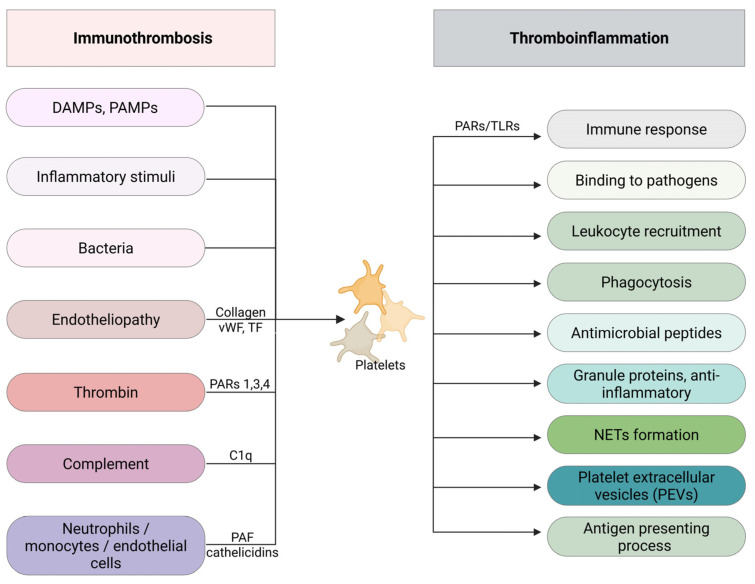
A figurative representation of platelets’ cornerstone role in immunothrombosis and thromboinflammation. Platelets in sepsis are activated by multiple triggers, such as DAMPs, PAMPs, inflammatory stimuli, bacteria, endothelial injury, thrombin, complement and immune cells. As a result, activated platelets reinforce multiple aspects of immune response, like pathogen binding, leukocyte recruitment, phagocytosis, NET formation, antigen presenting process and excretion of antimicrobial substances. Abbreviations: DAMPs, danger-associated molecular patterns; NETs, neutrophil extracellular traps; PAF, platelet activating factor; PAMPs, pathogen-associated molecular patterns; PARs, protease-activated receptors; PEVs, platelet extracellular vesicles; TF, tissue factor; TLRs, Toll-like receptors; VWF, von Willebrand Factor. Created in BioRender. Fortis, S. (2026) https://BioRender.com/0xvyj5u. Agreement number: RV29GQWGLE.

**Figure 3:**
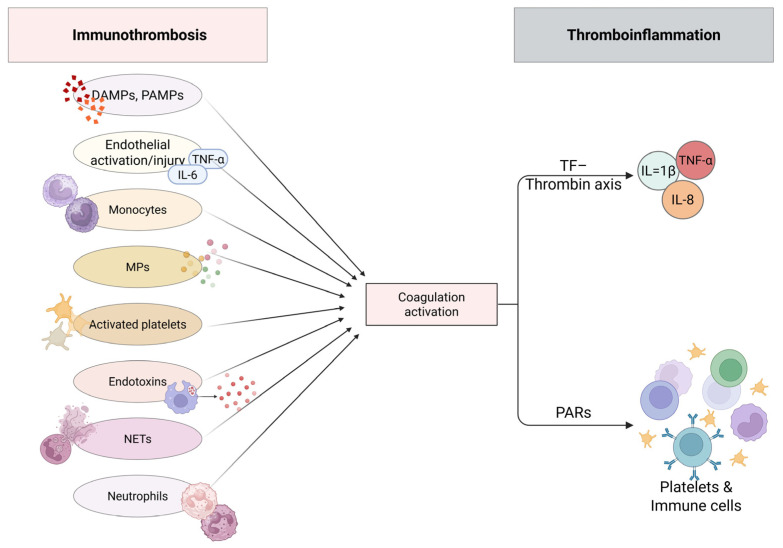
The dynamic interplay between immune response and coagulation activation in neonatal sepsis. DAMPs, PAMPs, pro-inflammatory cytokines, endothelial damage, monocytes, activated platelets, MPs, endotoxins, neutrophils and NETs activate the coagulation process. Coagulation activation enhances the production of IL-1b, IL-8 and TNF-α, while clotting factors regulate inflammation by binding to PARs of immune cells, leading to proteolytic cleavage of PARs and finally modulation of the immune response. Abbreviations: DAMPs, danger-associated molecular patterns; IL-1b, Interleukin-1b; IL-6, Interleukin-6; IL-8, Interleukin-8; MPs, microparticles; NETs, neutrophil extracellular traps; PAMPs, pathogen-associated molecular patterns; PARs, protease-activated receptors; TF, tissue factor; TNF-α, tissue necrosis factor-α. Created in BioRender. Fortis, S. (2026) https://BioRender.com/0xvyj5u. Agreement number: LF29GQWQGD.

Sepsis is often accompanied by deranged coagulation, ranging from subtle laboratory abnormalities to disseminated intravascular coagulation (DIC). Coagulation abnormalities constitute an important and independent predictor of clinical outcome in patients with sepsis [101]. Coagulation and inflammation are tightly connected, and it is likely that platelets, as well as the vascular endothelium, represent the main “bridge” between the two processes, leading to the new concept of immunothrombosis or thromboinflammation. This principle was further reinforced during the COVID-19 pandemic, as venous thromboembolism emerged as a serious complication, occurring despite prophylactic anticoagulation and contributing significantly to morbidity and mortality [102,103,104,105]. Additionally, growing evidence suggests that immunothrombosis is present in certain neonatal diseases, such as necrotizing enterocolitis and neonatal arterial ischemic stroke [106]. During the early phase of sepsis, the release of cytokines triggers coagulation, leading to a procoagulant state and subsequently to the development of intravascular fibrin deposition and multi-organ involvement. Bleeding may occur later in parallel, because clotting factors and natural inhibitors are depleted [107,108]. The conceptual role of immunothrombosis and thromboinflammation is demonstrated in Figure 2 and Figure 3.

Pathogen-associated molecular patterns (PAMPs), which represent cell fragments of pathogens, derived from the pathogen’s membrane, glycoproteins and nucleic acid, play a central role in SIC. Pathogen-associated molecular patterns (PAMPs) trigger both the innate immune response and the coagulation process. Another major component of SIC is represented by danger-associated molecular patterns (DAMPs), which are host-derived biomolecules released in stress, able to activate innate immunity, as well as procoagulant signaling. Monocytes and macrophages are activated by PAMPs and DAMPs to release cytokines and chemokines, which further promote neutrophil, platelet and endothelial activation. Additionally, activated monocytes deliver extracellular vesicles that contain TF and phosphatidylserine on their surfaces [109,110].

### 3.1. The Role of Vascular Endothelium in SIC

Vascular endothelium is an integral part of the human homeostatic mechanism, responsible for the maintenance of vascular tone, the permeability of fluids, nutrients and metabolic components, while it also acts as a barrier against toxins and microorganisms and exerts distinct anti-inflammatory and anti-coagulant action [111,112,113]. The endothelial glycocalyx is degraded under septic conditions due to the release of various metalloproteases, heparanase, hyaluronidase, lysosome-related organelles, reactive oxygen species (ROS), elastase and thrombin [114,115]. Overactivation of the sympathoadrenal system in septic shock also leads to glycocalyx injury and damage [116]. Structural derangement of endothelial glycocalyx is associated with endothelial functional disturbance, since many of the implicated proteins such as AT and complement regulatory proteins bind to glycocalyx heparan sulfate binding regions [117]. Moreover, the invasion of pathogens and the associated activation of the complement system in sepsis result in endothelial injury, and the subsequent endotheliopathy activates both the inflammatory pathway and the release of cytokines, as well as the microthrombi pathway, through expression of ULVWF and platelet activation [104]. Furthermore, glycocalyx damage results in enhanced leukocyte rolling and adhesion, loss of vascular integrity and tone, as well as activation of the coagulation cascade. The anticoagulant function of endothelium, exerted through AT, FIX and FX, is also disrupted [117]. Additionally, the endothelium surface molecules’ expression is altered, involving VWF, TF and adhesion molecules. Particularly, adhesion molecules, such as Intercellular Adhesion Molecule-1 (ICAM-1), Vascular Cell Adhesion Molecule-1 (VCAM-1), and E-selectin are implicated in the adhesion of monocytes, neutrophils, and platelets to vascular endothelium, leading to microthrombi formation. Angiopoietin-2 and PAI, expressed by endothelial cells, also regulate inflammation and coagulation [115,118]. Glycocalyx injury also leads to capillary leak syndrome (CLS), a recently well-described complication in neonatal septic syndromes. Capillary leak syndrome (CLS) is characterized by generalized edema, hypovolemia, hemodynamic instability and tissue hypoperfusion due to excessive fluid shift from the intravascular into the extravascular space [115,119]. Finally, deranged endothelium interacts with platelets activated in sepsis, further enhancing thromboinflammation [120].

### 3.2. The Role of Platelets in SIC

Platelets play a key role in SIC. In invertebrates, thrombocytes and phagocytes are not distinct, and hemocytes participate in innate immune response and clot formation. Evolutionarily, platelets arise from hemocytes, so it is rational to consider platelet as “another leucocyte”, involved substantially in inflammation processes [121,122,123]. Moreover, platelets represent a major component of innate immunity, since intravascular clot formation halts the dissemination of pathogens [124,125]. Inflammatory stimuli trigger platelets to express P-selectin on their surface, establishing the adhesion of platelets to leukocytes, as well as platelet aggregation [126]. What is more, thrombin generated by coagulation activation upregulates platelets through protease-activated receptors (PARs) 1, 3 and 4. The endothelium is activated in sepsis by proinflammatory cytokines and leukocytes, resulting in a prothrombotic condition [127]. A unifying term of “shock-induced endotheliopathy” has been introduced, in order to clarify the endothelial injury by endogenous catecholamines released in shock [128]. Endothelial activation during sepsis leads to collagen, VWF and TF exposure. Collagen and VWF bind to Platelet Glycoprotein VI (GPVI) and Platelet Glycoprotein Ib–IX–V complex (GPIb–GPIX–GPV) receptors, while TF induces the coagulation process and thrombin production, leading to further platelet activation and aggregation, as well as recruitment of immune cells. The complement system is also activated in bacterial infections, which further augments platelet function through the C1q receptor (C1qR) [129]. Furthermore, platelets are activated by proinflammatory mediators, such as platelet activating factor (PAF) produced by neutrophils, monocytes and endothelial cells, while neutrophils trigger platelets by the released antimicrobial cathelicidins [121,124] (Figure 2).

Moreover, platelets are activated by several bacteria and viruses directly or indirectly, through plasma proteins such as fibrinogen, VWF, complement and immunoglobulins. Furthermore, bacterial invasion augments the expression of the GPIIb/IIIa receptor (integrin αIIbβ3) on the activated platelet surface, as demonstrated in cases of *Staphylococcus aureus* and *Escherichia coli* infection, suggesting direct platelet activation by bacteria [130,131,132]. Other platelet receptors involved in platelet–bacteria interaction are GPIbα, PAR1, C1qR and Toll-like (Toll-like receptors, TLRs) [129,132]. Furthermore, *Staphylococcus aureus* virulence is strongly attributed to α-toxin, which has an additional negative effect by binding ADAM10 (a disintegrin and metalloprotease 10), leading to platelet and neutrophil upregulation and aggregation, subsequently resulting in organ damage during sepsis [133].

Activated platelets release platelet extracellular vesicles (PEVs), which play a pivotal role in intracellular communication via substance release and exchange. Platelet extracellular vesicles (PEVs) in sepsis take part in the control of coagulation and inflammation processes, as they exert 50–100 times greater procoagulant activity compared to activated platelets, while they also recruit leukocytes and excrete chemokines [132].

Platelet activation during sepsis is strongly associated with an anti-inflammatory effect, as they recognize inflammatory mediators, DAMPs, PAMPs and leukocytes [134]. Initially, human platelets express PARs, specifically PAR1 and PAR4, which interact with TLRs, thus modulating the immune response [135]. Platelets demonstrate clear antibacterial action by releasing platelet antimicrobial peptides (PMPs) or defensins [129]. Moreover, platelets’ TLRs promote immune reaction by binding to pathogens, while they induce leukocyte recruitment [30,124]. Additionally, activated platelets form complexes with leukocytes, facilitating bacterial phagocytosis [30]. The same complexes activate neutrophils to release neutrophil extracellular traps (NETs), which entrap bacteria, viruses, fungi and protozoa [136,137]. Additionally, platelets’ granules contain potent proteins, which exert clear anti-inflammatory action [121]. Moreover, Kuppfer cells in the liver sinusoids, the largest population of tissue macrophages in the body, interact with platelets in order to remove methicillin-resistant *Staphylococcus aureus* (MRSA) from the bloodstream [138]. Furthermore, platelets exert cytoprotective action during inflammation; for example, platelets inhibit lung epithelial apoptosis in pneumonia. Finally, platelets take part in the antigen presenting process [124]. Despite the impactful involvement of platelets in immune response, overactivation of them results in a hyperinflammatory reaction, through vascular-occluding thrombi and cytotoxic action of platelets and their microparticles (MPs), leading to organ damage [129] (Figure 2).

Thrombocytopenia is a common feature of neonatal sepsis, particularly in infections caused by Gram-negative bacteria or fungi. A low platelet count is associated with increased morbidity and mortality. The main mechanism leading to thrombocytopenia is increased consumption of platelets during clot formation, while platelets are also consumed into complexes with leukocytes [121,139]. However, neonatal sepsis at the same time upregulates thrombopoietin (TPO) production, and increased levels of megakaryocyte progenitors are found, leading to a hypercoagulable state in the early stages of sepsis. This response, however, is blunted in cases of Gram-negative neonatal sepsis, suggesting relatively diminished platelet production [140].

Functional platelet disorders are also observed in sepsis, as infection and concomitant acidosis impair platelet hemostatic function. Additionally, inflammatory mediators such as Interleukin-6 (IL-6) have been shown to stimulate platelet production from megakaryocytes; however, the newly released platelets are more thrombogenic [129]. Finally, septic preterm neonates have demonstrated impaired platelet adhesion in comparison to term neonates, mainly due to a functional deficiency, rather than quantitative or qualitative disorders of VWF [141,142].

### 3.3. Secondary Hemostasis in SIC

Secondary hemostasis is triggered by pro-inflammatory cytokines such as IL-6 and endothelial damage in a TF-dependent manner, leading to full activation of the coagulation “cascade” [139]. Moreover, monocytes are activated by tumor necrosis factor-α (TNF-α) and IL-6 leading to further stimulation of platelets and TF-dependent production of thrombin. Activated platelets, macrophages and endothelial cells release MPs, which take part in coagulation activation by binding to TF and VWF [121,129,143,144]. Activated platelets permit the assembly of clotting factors, leading to a severalfold increase in the generation of thrombin [101]. Neutrophils upon activation release NETs, which enhance coagulation due to their negative charge, besides their antibacterial and antiviral action. Delivery of TF is another prothrombotic property of NETs, ultimately leading to massive clot formation [121,135]. Another implication of neutrophils in coagulation activation is the expression of TF by them. Moreover, bacterial endotoxins trigger coagulation by binding to monocytes and endothelial cells, resulting in cytokine production and TF-mediated coagulation activation [110] (Figure 3).

**Figure 4:**
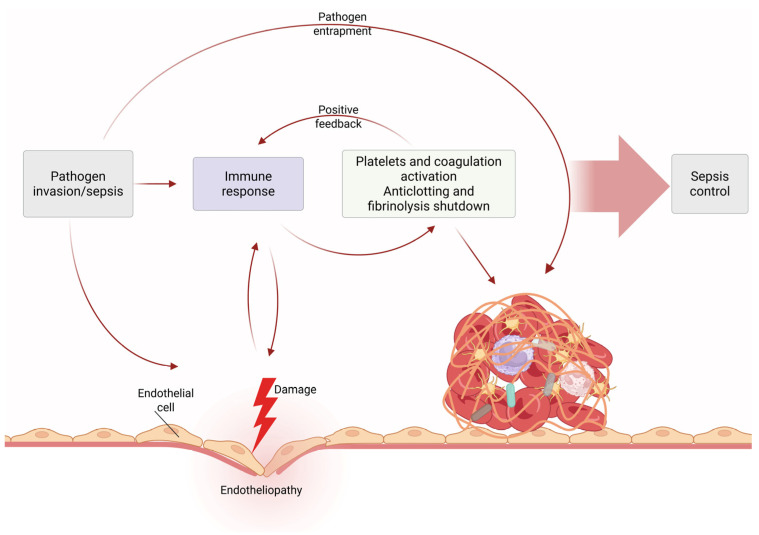
Pathogen invasion and the activation of sepsis cascade result in endothelial damage and upregulation of immune response, which both activate the coagulation process. Coagulation activation in turn further enhances the immunologic response. Clotting enhancement and the associated shutdown of anticlotting and fibrinolytic mechanisms under septic conditions result in thrombus formation, pathogen entrapment into this and ultimately sepsis control, clarifying that the strong crosstalk between immune and coagulation pathways exerts a significant effect on human host defense. Created in BioRender. Fortis, S. (2026) https://BioRender.com/0xvyj5u. Agreement number: RV29GQWZSP.

The triggered hemostatic mechanism and the immune response are tightly inter-related. Inducible TF is mainly expressed by monocytes and macrophages. Monocytes are further stimulated by platelets and granulocytes to express TF. This reaction enhances the production of Interleukin-1b (IL-1b), Interleukin-8 (IL-8) and TNF-α [101]. Furthermore, coagulation factors regulate inflammation by binding to PARs of immune cells. The subsequent proteolytic cleavage of PARs by activated coagulation factors results in modulation of the immunologic response (Figure 3). This model further verifies the assumption that inflammation activates coagulation and coagulation further upregulates the inflammatory response [139,144]. Nevertheless, coagulation represents a significant part of innate immunity and a host-defending mechanism, since clot formation tends to restrict the dissemination of pathogens into the circulation [121] (Figure 4).

### 3.4. Fibrinolysis in SIC

Regarding fibrinolysis, certain functional disorders take place in sepsis. Initial stages of infection are characterized by an upregulation of fibrinolytic activity due to enhanced action of tPA. As the disease progresses, a fibrinolysis shutdown takes place due to increased PAI release by endothelial cells [81,110]. Studies based on TNF administration in humans demonstrated an early, though transient, activation of fibrinolysis, which evolved into fibrinolytic inhibition by PAI [145]. It has been demonstrated that a functional mutation in the PAI-1 gene (the 4G/5G polymorphism) is related not only to the altered levels of PAI but also to the clinical outcome of meningococcal septicemia [146]. Increased concentrations of thrombin in sepsis result in activation of TAFI, a significant inhibitory mechanism of fibrinolysis in sepsis. Moreover, APC induces fibrinolysis through PAI inhibition and suppressed production of TAFI. Endothelial damage in sepsis impairs activation of PC to APC, leading to diminished fibrinolytic activity [147]. However, apart from plasmin, neutrophil elastase also contributes to fibrinolysis by degrading fibrin through a mechanism distinct from that of plasmin [148]. Nevertheless, fibrin formation in sepsis is ultimately dysregulated, leading to significant organ damage and a clear association with increased mortality [149].

### 3.5. The Anticlotting System in SIC

Sepsis-induced coagulopathy (SIC) is characterized by a clear downregulation of anticlotting mechanisms. Endothelial integrity is interrupted in sepsis, as the endothelial glycocalyx structure is deranged, thus impairing endothelium–neutrophil–platelet interaction, leading to thrombus formation. Injured endothelial glycocalyx expresses glycosaminoglycans, such as heparan sulfate, at a lower level, further contributing to suppressed endothelial anticoagulant properties [139]. Reduced ADAMTS-13 levels have been reported across adult, pediatric, and neonatal sepsis and are associated with impaired ULVWF cleavage and increased thrombogenicity. The magnitude of ADAMTS-13 reduction has been associated with disease severity and poor prognosis, likely due to thrombotic microangiopathy and subsequent end-organ damage [2,150,151,152].

Antithrombin (AΤ) is also consumed during thrombin’s inhibition process, while it is inactivated by neutrophil elastases found at increased levels in the case of infection [110,153,154]. Thrombomodulin (TM) also inactivates thrombin and is downregulated by TNF-α [143]. Furthermore, the coagulation system is less susceptible to its natural inhibitors (AT, PC, PS and TFPI) in the case of inflammation. Liver function is compromised in sepsis, as reflected by raised values of the relevant enzymes (alanine transaminase and aspartate transaminase). Liver dysfunction probably leads to reduced levels of AT, PC and PS, which may serve as an adjuvant tool in the early diagnosis of sepsis. Consumption coagulopathy is another contributing factor, as well as altered vitamin K metabolism in regard to PC and PS [154,155]. El Beshlawy et al. compared 30 septic term newborns with 30 age-matched controls and documented a significant decrease in AT, PC, and PS in 100% of cases (*p* < 0.001). In the sepsis group, 33% of the neonates developed DIC and died, and a significant proportion developed many other neonatal morbidities, such as necrotizing enterocolitis and intracranial hemorrhage. Protein C (PC) levels were most markedly reduced in those who developed DIC and died [107]. Additional studies have shown statistically significantly lower PC and AT levels in non-surviving vs. surviving septic neonates [156,157]. Moreover, C4b-binding protein (C4b-BP), the binding protein of PS, represents a complement inhibitor and is increased in the case of infection, resulting in reduced levels of free and active PS [13,158]. Reduced levels of natural inhibitors in neonatal sepsis have also been reported in our previous study [2].

Furthermore, AT and PC clearly exert anti-inflammatory properties besides their antithrombotic action. Activated protein C (APC) downregulates the production of TNF-α, IL-1b, IL-6 and IL-8 by blocking monocytes and macrophages [139]. Protein C (PC) also inhibits the interaction between neutrophils and endothelium and the complex interplay between PC, Endothelial Protein C Receptor (EPCR) and neutrophils comprise another component of PC’s anti-inflammatory action [159]. Substitution therapy with recombinant human protein C (rhAPC) has been widely investigated in randomized controlled trials, with patients receiving rhAPC demonstrating markedly lower D-dimer and IL-6 levels, consistent with both anticoagulant and anti-inflammatory effects [160]. Although rhAPC was approved by the FDA and EMA, later RCTs failed to demonstrate a survival benefit in septic shock, resulting in its withdrawal. Likewise, AT administration showed no benefit in randomized trials [121,161,162]. In regard to neonates, the use of rhAPC in the management of neonatal sepsis is discouraged, due to the substantial risk of intraventricular hemorrhage in newborns and a lack of clear benefit in children [159,163].

It is worth noting that AT and PC exert significant anti-inflammatory effects, partly due to their anticoagulant activity. As mentioned above, thrombin and other coagulation factors further stimulate the inflammatory response; therefore, their inhibition by AT and PC results in the downregulation of inflammation [162,164]. Antithrombin (AT) also exerts anti-inflammatory properties independently of its anti-coagulant action. Direct antimicrobial effects of AT through binding and perforation of bacterial cell walls have been recently introduced. Besides the antibacterial effect, AT acts against fungi, viruses and parasitic infections [165]. More thoroughly, AT enhances prostacyclin release by endothelial cells, which further inhibits platelet aggregation and activation, binding of neutrophils on blood vessel walls and production of cytokines and chemokines by endothelial cells. Additionally, AT interacts directly with leukocytes and lymphocytes and inhibits their interplay with endothelial cells, while the direct binding of AT to certain cellular receptors inhibits the pro-inflammatory process. Proteolytic cleavage of AT leads to release of antibacterial peptide fragments [165]. The broad anticoagulant and anti-inflammatory actions of AT may have clinical applications in conditions characterized by both coagulation and inflammation, such as pregnancy, sepsis, and DIC [162].

### 3.6. DIC in Neonatal Sepsis

Disseminated intravascular coagulation (DIC) has been traditionally described as a unique clinical entity, where bleeding and thrombosis exist simultaneously. Excessive coagulation activation leads to microthrombus formation and consumption of platelets and clotting factors, resulting in a hemorrhagic state [127,144]. Available evidence on the exact incidence and subsequent impact of DIC on the outcome of term and preterm neonates is lacking, but previous studies demonstrate an alarming prevalence of DIC in neonates who die in neonatal intensive care units (NICUs) [45]. The prognostic significance of DIC is supported by paraphrasing its abbreviation as “Death Is Coming” [166]. Disseminated intravascular coagulation (DIC) is often subclinical during neonatal age. Dairaku et al. identified histopathological evidence of DIC in 24 of 201 consecutive neonatal autopsies, including microthrombi in three or more organs. Nevertheless, only one case showed clinical signs of DIC, highlighting its underdiagnosis in early life [167]. The pathophysiology of DIC should always be considered in conjunction with the underlying condition, which should be first treated, to manage DIC effectively. Nevertheless, in overt DIC, coagulopathy may persist despite correction of the precipitating cause and may require targeted therapy. In newborns, hypoxic–ischemic injury and sepsis are the most common triggers [45]. Sepsis-induced coagulopathy (SIC) represents a herald of DIC, and its early identification is crucial, in order to implement a prompt therapeutic approach and alleviate the unfavorable impact of DIC [168]. The coagulation cascade is activated in SIC, though the consumption of platelets, clotting and clotting factors is not yet present [169].

Appropriate diagnostic criteria of SIC and DIC have been implemented in adults. Disseminated intravascular coagulation (DIC) criteria integrate thrombocytopenia, PT prolongation, elevated D-dimers, low fibrinogen levels and Sequential Organ Failure Assessment (SOFA) score [170]. The aforementioned developmental changes of hemostatic parameters, such as the normal elevation of D-dimers, make these criteria less useful in neonates. However, SIC represents an earlier phase of hemostatic derangement, during which PAI levels are increased, fibrinolysis is downregulated and fibrinogen levels are within normal limits [171]. Thus, SIC criteria in adults integrate only low platelet count, PT prolongation and SOFA score, and their implementation in neonates may be more suitable [172]. Indeed, recent studies have implemented a pediatric SIC score modified from adults and tested its diagnostic and prognostic usefulness. The pediatric SIC score demonstrated an area under the receiver operating characteristic curve (AU-ROC) of 0.716 (optimal cut-off > 3) for predicting 28-day mortality in sepsis, an AU-ROC of 0.845 (optimal cut-off > 3) for predicting non-overt DIC and an AU-ROC of 0.901 (best cut-off > 4) for predicting overt DIC [173]. Similarly, another study conducted by Jhang and Park investigated the utility of a pediatric SIC score, based on the pediatric SOFA score, PT and D-dimers. The particular pediatric SIC score had an AU-ROC of 0.771 for predicting 28-day mortality, while it established the diagnosis of SIC in 50.4% of pediatric patients with septic shock [174]. However, similar studies are lacking in the neonatal population, probably due to the inherent difficulties of blood sampling and conducting laboratory investigations in critically ill neonates. Although age-appropriate SOFA scores for children and neonates have been validated, their widespread employment in everyday clinical practice is not yet apparent [175,176,177]. Moreover, the most recent pediatric sepsis definition does not incorporate pediatric and neonatal SOFA score [178,179].

Future large-scale studies of SIC/DIC scoring systems in neonates are imperative. Besides conventional coagulation tests, the uniform and consistent use of viscoelastic assays in NICUs, which require small blood volumes and are clearly superior in assessing the hemostatic mechanism, could likely promptly identify the switch from compensated (SIC) into decompensated (DIC) coagulation and guide appropriate therapeutic interventions.

### 3.7. Further Features of “Immunothrombosis”

Other examples of the strong interplay between coagulation and inflammation are the implications of the contact and the complement systems. Factor XII (FXII) of the contact system is activated by interaction with negatively charged surfaces, in addition to bacterial, fungal and viral surfaces, and subsequently activates the proinflammatory kallikrein–kinin system as well as the intrinsic pathway of coagulation [180]. Moreover, the complement system triggers platelets and coagulation, while platelets are involved in complement activation [181]. Relatively underestimated is the role of erythrocytes in the “immunothrombosis” process. Erythrocytes are involved in thrombus formation and exert procoagulant activity through meizothrombin expression, an intermediate between prothrombin and thrombin. Erythrocytes also entrap and kill bacteria under high velocities of blood flow, contributing to the host defense mechanism [121].

### 3.8. Pathogen-Specific Coagulation Disorders

It is well established on clinical grounds and in everyday practice of neonatology that Gram-negative septicemia is associated with increased morbidity and mortality compared to Gram-positive sepsis, though the clinical impact of Gram-positive infections is also substantial and cannot be overlooked. Many studies have concluded that neonatal sepsis caused by Gram-negative bacteria is associated with a tendency towards higher levels of inflammatory markers, as well as increased severity and mortality, compared to Gram-positive sepsis [182,183,184,185]. Recently, Sokou et al. investigated the ROTEM parameters in neonates with Gram-negative and Gram-positive septicemia and elucidated a substantially hyper-coagulable state in Gram-positive rather than Gram-negative neonatal sepsis. Notably, neonates with Gram-negative sepsis demonstrated a more hypocoagulable state, associated with a greater risk of hemorrhage. Moreover, the study revealed a greater incidence of thrombocytopenia in Gram-negative sepsis, probably due to increased activation of platelets, their incorporation into the host defense mechanism and ultimately their consumption upon DIC development [185]. These findings are in line with additional studies, demonstrating lower platelet counts in Gram-negative neonatal sepsis, as well as diminished platelet production and thrombopoietin excretion in very-low-birth-weight neonates, a problem frequently encountered in clinical practice [182,186]. Similarly, Gialamprinou et al. studied the ROTEM parameters, conventional coagulation tests and platelet function sequentially in preterm neonates suffering from Gram-positive septicemia and compared the results to healthy counterparts. It is noteworthy that the research team excluded the cases of Gram-negative sepsis, due to overwhelming clinical course, complicated by fatal DIC, severe thrombocytopenia and Grade III–IV intraventricular hemorrhage. ROTEM assay exhibited hypercoagulability and decreased fibrinolytic activity, while conventional coagulation tests demonstrated hypocoagulability in the sepsis group compared to healthy one. Septic neonates exhibited thrombocytopenia and reduced platelet activation. Glycoprotein (GP) expression was increased in the septic group and appeared to be age-dependent. The study highlights a significant discrepancy between viscoelastic assays and conventional coagulation tests, which may lead to markedly different clinical decisions, potentially with detrimental effects on outcomes [187].

Moreover, Sokou et al. investigated the impact of neonatal Candida infections on ROTEM parameters and demonstrated a hypocoagulable state during the early stages of the disease. Additionally, a strong correlation was found between hemostatic derangement and disease severity, as evaluated using neonatal SOFA (nSOFA), Modified Neonatal Multiple Organ Dysfunction (NEOMOD) and Neonatal Bleeding Assessment Tool (NeoBAT) scores. The particular results clearly demonstrate that the severity of the underlying infection strongly affects the coagulation disturbance. The causality is probably multifactorial, including greater activation of coagulation factors by fungal proteases, impaired peripheral circulation and reduced tissue perfusion [188].

Additionally, specific therapeutic agents have been associated with greater hemostatic alteration, such as b-lactam antibiotics, aminoglycosides and anti-fungal drugs. For example, the N-methylthiotetrazole (NMTT) side chain of cephalosporins can downregulate the activation of FII, FVII, FIX and FX in the liver and lead to hypoprothrombinemia.

Thus, it could be speculated that the specific treatment also affects the coagulation process, not only the septic cascade per se [189,190,191,192,193].

On the other hand, certain studies on viscoelastic tests in septic neonates have shown a hypocoagulable state in neonatal sepsis, irrespectively of the underlying pathogen [79,188,194]. Despite the conflicting results between the studies, viscoelastic assays represent a valuable tool in assessing coagulation abnormalities associated with neonatal sepsis, while they can also serve as an adjuvant method to predict outcomes [82].

### 3.9. Beneficial Role of SIC

The deranged coagulation in sepsis is not certainly harmful; on the contrary, it is a strong host defense mechanism, as demonstrated in Figure 4. It has been demonstrated that adult septic patients carrying the FV Leiden mutation, an essential thrombogenic condition, had improved prognosis in comparison to patients negative to this specific mutation. This finding was verified by animal experiments [195]. Studies have indicated that the hemostatic system also contributes to mammalian innate defense, since the activation of coagulation factors eventually leads to bacterial entrapment inside the fibrin clot and generation of small antibacterial peptides [185,196]. Furthermore, intravascular thrombi induce innate immune response, consequently leading to recognition of offending microorganisms, restriction of their spread and finally their destruction [181,197]. Particularly, clot formation acts by preventing the widespread dissemination of pathogens, while fibrin, fibrinogen and their degradation products support the recruitment of leukocytes [135]. Moreover, the activation of the contact system by pathogens is a strong contributing factor in the innate immune response. For example, in cases of *Streptococcus pyogenes* infection, the contact system is activated early, resulting in clot formation and entrapment of bacteria inside the clot. However, the late stage of infection is associated with plasmin upregulation, leading to fibrin clot lysis and subsequent release of bacteria [198]. Despite the beneficial impact of the coagulation process in immune response, its overactivation can lead to life-threatening sequelae, such as thrombosis, DIC, bleeding and tissue damage [135].

## 4. Discussion and Practical Recommendations

Coagulation abnormalities in neonatal sepsis are often obscure and subclinical, especially early in the disease process. All clinicians that take care of term and preterm newborns should have a high index of suspicion in identifying and promptly treating sepsis and its associated impact on hemostasis. Conventional and, if feasible, viscoelastic coagulation tests should be universally employed in all septic neonates, and age-specific reference ranges should be used for their interpretation. An ideal assessment would include sequential testing that would reflect the dynamic nature of coagulation abnormalities, ranging from SIC to overt, decompensated DIC. Targeted transfusions of blood products are often essential and life-saving; however, they are associated with increased mortality, fluid overload, hemodynamic instability and certain complications; thus, they should be used with caution and be justified. Transfusions of platelets and fresh-frozen plasma should be guided by the bleeding risk, rather than absolute numerical values, and their liberal prophylactic administration is not supported. Besides the increased awareness of neonatologists, a multidisciplinary approach is highly recommended, including neonatologists, hematologists and transfusion specialists. Further large-scale studies on neonatal SIC/DIC scores and their utility in neonatal sepsis are of paramount importance, and research teams should focus towards this direction. In accordance with Hippocrates’ statement that “prevention is better than cure”, probably the most useful tool in the fight against coagulation abnormalities in neonatal sepsis is their prevention. All NICUs should establish specific protocols on infection control, while antibiotic stewardship programs should be implemented, in order to decrease the burden of the disease.

## 5. Conclusions

In summary, the coagulation system in healthy neonates exerts unique features, preserving a delicate balance and eventually eliminating the risk of bleeding or thrombosis. Developmental aspects of coagulation are essential for normal human development, and derangement of them in certain pathological situations can lead to a vast array of unfavorable consequences. Sepsis-induced coagulopathy (SIC) in neonates is a well-recognized condition, associated with a prothrombotic tendency in early disease states, followed by consumption of clotting and anticlotting factors. Hemostatic derangement in neonatal sepsis exerts a significant clinical impact, and clinicians taking care of term and preterm neonates should have a high index of suspicion in order to promptly recognize and effectively treat this condition. Growing evidence highlights the superiority of viscoelastic assays in diagnosing SIC in comparison to conventional coagulation tests, which can have misleading results. Sepsis-induced coagulopathy (SIC) reflects a complex interplay between protective and deleterious mechanisms within the host response. In the initial phase, activation of the coagulation cascade plays a critical immunological role, contributing to pathogen containment and limiting infection dissemination. However, when this response becomes dysregulated or excessive, it evolves into a pathological process characterized by widespread microvascular thrombosis, endothelial injury, and consumption of platelets and coagulation factors, ultimately progressing to DIC. This cascade impairs organ perfusion, resulting in ischemia and multi-organ dysfunction. Simultaneously, depletion of coagulation components may manifest as either a thrombotic or bleeding phenotype, further worsening clinical outcomes. Thus, although coagulation is inherently protective during the early stages of sepsis, its overactivation highlights the dual nature of sepsis-induced coagulopathy, acting as both friend and foe, with profound implications for patient prognosis.

## Data Availability

No new data were created or analyzed in this study.

## Abbreviations

The following abbreviations are used in this manuscript:

ADAM10	A Disintegrin and Metalloprotease 10
ADAMTS-13	A Disintegrin and Metalloprotease with Thrombospondin Type-1 Motifs 13
APC	Activated Protein C
APTT	Activated Partial Thromboplastin Time
AT	Antithrombin
AU-ROC	Area Under the Receiver Operating Characteristic Curve
C1qR	C1q Receptor
C4b-BP	C4b-Binding Protein
CFT	Clot Formation Time
CT	Clotting Time
DAMPs	Danger-Associated Molecular Patterns
DIC	Disseminated Intravascular Coagulation
EPCR	Endothelial Protein C Receptor
EXTEM	Extrinsic Pathway Thromboelastometry Assay
FII	Factor II (Prothrombin)
FIIa	Activated Factor II (Thrombin)
FV	Factor V
FVa	Activated Factor V
FVII	Factor VII
FVIIa	Activated Factor VII
FVIII	Factor VIII
FVIIIa	Activated Factor VIII
FIX	Factor IX
FIXa	Factor IXa
FX	Factor X
FXa	Activated Factor X
FXI	Factor XI
FXIa	Activated Factor XI
FXII	Factor XII
FXIIa	Activated Factor XII
FXIII	Factor XIII
GP	Glycoprotein
GPIbα-GPIX-GPV	Platelet Glycoprotein Ib–IX–V Complex
GPIIb/IIIa	Platelet Glycoprotein IIb/IIIa (Integrin αIIbβ3)
HCII	Heparin Cofactor II
HMWK	High-Molecular-Weight Kininogen
IL-1b	Interleukin-1 Beta
IL-6	Interleukin-6
IL-8	Interleukin-8
MCV	Mean Corpuscular Volume
MPs	Microparticles
MRSA	Methicillin-Resistant Staphylococcus aureus
NETs	Neutrophil Extracellular Traps
NICU	Neonatal Intensive Care Unit
PAF	Platelet Activating Factor
PAI	Plasminogen Activator Inhibitor
PAMPs	Pathogen-Associated Molecular Patterns
PARs	Protease-Activated Receptors
PC	Protein C
PEVs	Platelet Extracellular Vesicles
PK	Prekallikrein
PMPs	Platelet Antimicrobial Peptides
PS	Protein S
PT	Prothrombin Time
PZ	Protein Z
RBC	Red Blood Cell
RDS	Respiratory Distress Syndrome
rhAPC	Recombinant Human Activated Protein C
ROTEM	Rotational Thromboelastometry
SIC	Sepsis-Induced Coagulopathy
SOFA	Sequential Organ Failure Assessment
TAFI	Thrombin Activatable Fibrinolysis Inhibitor
TEG	Thromboelastography
TF	Tissue Factor
TFPI	Tissue Factor Pathway Inhibitor
TM	Thrombomodulin
TNF-α	Tumor Necrosis Factor Alpha
tPA	Tissue Plasminogen Activator
TPO	Thrombopoietin
TLRs	Toll-Like Receptors
TxA2	Thromboxane A2
ULVWF	Ultra-Large von Willebrand Factor
uPA	Urokinase Plasminogen Activator
VWF	Von Willebrand Factor
ZPI	Protein Z-Dependent Protease Inhibitor
